# The role of cytokines as inflammatory mediators in preeclampsia

**DOI:** 10.11604/pamj.2015.20.219.5317

**Published:** 2015-03-10

**Authors:** Ifeoma Udenze, Casimir Amadi, Nicholas Awolola, Christian Chigozie Makwe

**Affiliations:** 1 Department of Clinical Pathology, College of Medicine, University of Lagos, Lagos, Nigeria; 2 Department of Medicine, College of Medicine, University of Lagos, Lagos, Nigeria; 3 Department of Anatomic and Molecular Pathology, College of Medicine, University of Lagos, Lagos, Nigeria; 4 Department of Obstetrics and Gynaecology, College of Medicine, University of Lagos, Lagos, Nigeria

**Keywords:** Inflammation, cytokines, severe preeclampsia, pregnant women, hypertension

## Abstract

**Introduction:**

This study is to determine the concentrations of IL-6, TNF α, and C reactive protein (CRP) in women with severe preeclampsia, and compare with those of gestational age- matched normotensive pregnant women and to correlate CRP levels with markers of organ damage in women with preeclampsia.

**Methods:**

This was a case control study of fifty women with severe preeclampsia and fifty gestational age matched pregnant women with normal blood pressure. The women were drawn from The Antenatal Clinic of The Lagos University Teaching Hospital. Severe pre eclampsia was defined as systolic blood pressure ≥160 mmHg and/or diastolic blood pressure ≥110mmHg and ≥2+ of proteinuria. After obtaining an informed consent, each participant completed a structured questionnaire. The questionnaire sought information on socio-demographic and clinical data. From each participant, mid-stream urine was collected for urinalysis and culture, and blood sample was collected for biochemical analysis. Comparisons of continuous variables and categorical variables were done using the Student's t test and Chi square test respectively. Correlation analysis was used to determine the associations between variables. Statistical significance was set at P

**Results:**

The women were similar in their socio demographic characteristics. There was a statistically significant difference in the systolic blood pressure (p < 0.0001), diastolic blood pressure ( p < 0.0001), uric acid ( p < 0.0001), AST ( p < 0.0001), ALP ( p < 0.0001), creatinine ( p < 0.0013), GGT ( p < 0.005), IL 6 ( p < 0.021), CRP ( p < 0.0002), and TNF α ( p < 0.023), between the group with severe preeclampsia and the group with normal blood pressure. This study also reports a significant association between CRP and systolic blood pressure, diastolic blood pressure, uric acid AST and ALP (p

**Conclusion:**

The inflammatory cytokines, IL6, TNF α and CRP are elevated in severe preeclampsia and may mediate some of the clinical manifestations of the disorder. A role may exist for anti inflammatory agents in the management of women with preeclampsia.

## Introduction

A complex relationship exists between immunity, inflammation and disease. This relationship is played out in pregnancy, from the adaptation in immune responses necessary for the mother to tolerate the immunologically different foetus throughout the period of pregnancy to the role of inflammation in abnormal pregnancy outcome such as preeclampsia and pre term birth.

Immune adaptation in normal pregnancy has been associated with a decrease in the T helper 1 cytokines: interleukin 2 (IL2), interferon gamma (IF γ) and transforming growth factor beta (TGF β), which are involved in cellular immunity and mediate immune rejection of the foetus; and an increase in the T helper 2 cytokines: interleukins 4, 5, 6 and 13, which mediate humoral immunity, suppress cellular immunity and thereby prevent immune rejection of the foetus [1–3].

An abnormality in this delicate immunologic balance has been implicated in the pathology of preeclampsia [4]. The defective trophoblast invasion associated with preeclampsia may be as a result of some degree of immune intolerance [5, 6]. During vascular development of the placenta, cytotrophoblasts invade the uterine spiral arteries, replacing the endothelial layer. By the end of the second trimester, the uterine spiral arteries are lined exclusively by cytotrophoblast. The remodelling of the spiral arteries results in a low resistance arteriolar system with a dramatic increase in blood supply to the growing foetus [1]. In preeclampsia, there is limited invasion of the spiral arteries to only the superficial layers of the decidua. The failure of trophoblast invasion results in reduced uterine perfusion pressure and placental ischaemia [6]. The ischemic placenta may induce the release of bioactive circulating factors including pro inflammatory cytokines that may contribute to mediate the wide spread endothelial damage pathognonomic of preeclampsia [1]. The roles of the various cytokines in the pathology of preeclampsia has been studied by several authors, with conflicting reports on the serum concentrations of interleukins in preeclampsia [7–9]. Greer et al; [10] reported increased plasma concentration of IL-6, but normal concentration of IL-8 in preeclampsia while Olusi et al; [7] reported significantly lower IL-6 and IL-8 levels in preeclampsia compared to normal pregnancy. As a result of these controversies, we decided to measure the serum concentrations of IL-6, TNF α, and C reactive protein (CRP) in women with severe preeclampsia, and to determine if they differ from those of gestational age matched, normotensive, pregnant women. Also this study aims to determine the relationship, if any, between CRP levels and markers of organ damage (such as in the kidney and liver) in women with preeclampsia.

## Methods

### Subjects

The study took place during the period of July 2012 to August 2013. The cases and controls were drawn from women attending The Ante natal Clinic at Lagos University Teaching Hospital, Lagos Nigeria. This observational case control study consisted of fifty women with severe preeclampsia who were recruited as cases and fifty pregnant women with normal blood pressure who were recruited as controls.

**Case definition:** The cases consisted of pregnant women in whom a diagnosis of severe preeclampsia had been made. These women were in their third semesters of pregnancy. Severe preeclampsia was defined as systolic blood pressure ≥ 160 mmHg and/ or diastolic blood pressure ≥ 110mmHg and ≥ 2+ of proteinuria [11, 12].

**Control definition:** the controls were chronological and gestational age matched pregnant women whose blood pressures have remained normal during pregnancy; ≤120mmHg systolic blood pressure and ≤80mmHg diastolic blood pressure. Gestational age was calculated from the last normal menstrual period and/or by an early obstetric ultrasonography.

**Inclusion criteria:** the Inclusion criteria included absence of labour, absence of premature rupture of membranes and febrile illness in pregnancy.

**Exclusion criteria:** the exclusion criteria included multiple gestation, diabetes mellitus, sickle cell disease and HIV infection. Before recruitment, an informed consent was obtained from each study participant. The institutional review board/ Ethics committee of the Lagos University Teaching Hospital, Lagos, Nigeria, approved the study protocol.

### Materials and methods

Mid stream urine specimen and blood was collected for analysis. Specimen with positive urine culture and elevated white cell count, were excluded. Blood was centrifuged at 4000 RPM for 10 minutes and stored at -800C in aliquots till analysis. Freeze thaw cycles were avoided. Plasma creatinine, urea and uric acid were estimated from lithium heparin plasma using reagent from Biolabo laboratories, 02160, Maizy, France. Serum levels of aspartate amino transferase (AST), alanine amino transferase (ALT), alkaline phosphatise (ALP) and gamma glutamyl transferase (GGT) were estimated from lithium heparin plasma using reagents from Randox Laboratories Limited, Antrim, UK, BT 29 4QY. Spectrophotometric methods were employed for the biochemical analysis on semiautomatic biochemistry analyser BS3000P-Sinnowa Medical Science and Technology Company limited, Nanjing, China (211135).

Plasma levels of IL6, TNF α and CRP were determined from EDTA plasma using reagents from Biovendor Laboratories, 62100 Brno, Czech Republic by an enzyme linked immunoassay technique [13] on Acurex Plate Read - Acurex Diagnostics, Ohio, USA (419-872-4775)

### Statistical analysis

The data were analyzed using the IBM SPSS version 19.0 package. Independent student's t test was used to test the differences in the mean values for the continuous variables. Chi square test was used to test the differences in proportion of the catergorical variables. Correlation analysis was used to determine the association between variables. Statistical significance was set at p < 0.05.

## Results

The group with severe preeclampsia and the normotensive pregnancy control group did not differ in their socio demographic characteristics. Forty – nine percent (49%) of the group with severe preeclampsia were of the Yoruba tribe, forty –two (42%) were Igbo and nine percent (9%) were from other tribes. In the normotensive pregnancy control group, forty-eight percent (48%) were Yoruba, forty three percent (43%) were Igbo and nine percent (9%) were from other tribes. Ninety –eight percent (98%) of the group with severe preeclampsia were married and two percent (2%) were unmarried. In the normotensive pregnancy control group, ninety –five percent (95%) were married and five percent (5%) unmarried. Comparing educational status, five percent (5%) of the cases had only primary school education while twenty four percent (24%) and seventy one percent (71%) had secondary and tertiary education respectively. In the control group, twenty percent (20%), thirty one percent (31%) and forty nine percent (49%) of the women had primary, secondary and tertiary education respectively. The study participants did not differ in age distribution. The mean age of the cases was 31.90± 6.3 years and 32.33±5.8 years for the controls (p = 0.82). The mean gestational age of the cases was 30.95±3.79 weeks and 31.23±4.77 weeks for the controls (p = 0.84)

Table 1 shows the clinical and laboratory characteristics of the study participants. The inflammatory markers, IL6, TNF α and CRP were significantly higher in the group with severe preeclampsia. Table 2 shows correlation of CRP with markers of renal and liver damage in severe preeclampsia. There was an association between CRP and systolic blood pressure, diastolic blood pressure, uric acid, ALP and AST in women with severe preeclampsia. No such relationship was observed in women with normotensive pregnancy.

**Table 1:** Clinical and laboratory characteristics of the study participants

Characteristics	Severe Preeclampsia (N = 50), Mean± SD	Controls (N = 50), Mean± SD	P value
Age (years)	31.90±6.34	32.33±5.88	0.82
SBP (mmHg)	170.20±22.71	110.90±15.74	<0.0001+
DBP (mmHg)	100.90±37.74	71.38±6.37	<0.0001+
G. A (weeks)	30.95±3.79	31.23±4.77	0.84
BMI (kg/m^2^)	29.12±4.69	26.97±4.91	0.093
Creatinine(µmoles/L)	175.11±137.88	60.97±15.20	0.0013+
Uric acid(µmoles/L)	448.83±104.73	215.05±44.75	<0.0001+
Urea (mmoles/L)	3.95±2.84	3.74±7.57	0.91
ALT (U/L)	14.51±19.27	14.27±2.84	0.96
AST (U/L)	43.85±27.36	4.08±1.29	<0.0001+
ALP (U/L)	248.96±112.29	146.81±6.04	<0.0001+
GGT (U/L)	31.63±33.73	8.23±2.71	0.005+
IL 6 (pg/ml)	95.21±143.27	12.92±24.47	0.021+
CRP (mg/L)	44.98±37.50	6.97±13.06	0.0002+
TNF α (pg/ml)	44.80±37.52	8.15±23.24	0.023+

+ statistically significant

**Table 2:** Correlation of CRP with markers of organ damage in severe preeclampsia compared with normal pregnancy

Parameters	Severe Preeclampsia	Normal pregnancy
SBP	0.507+	0.039
DBP	0.519+	-0.066
Urea	0.035	-0.061
Uric acid	0.439+	0.045
Creatinine	0.305	-0.053
ALP	0.509+	-0.030
AST	0.384+	-0.210
ALT	-0.085	-0.410
GGT	0.276	-0.29

+ statistically significant

## Discussion

This study reports a statistically significant difference in the levels of the pro inflammatory cytokines, IL 6 and TNF α and in CRP levels in the women with severe preeclampsia, compared with women with normotensive pregnancy. Similar increases in the plasma levels of these markers were also reported by Teran et al; [14] and Hentschke et al; [15] who reported increased soluble IL6 receptors in preeclampsia. Ozier et al; [16] found no difference in the levels of IL6 and TNF α in preeclampsia. Afshari et al; [8] found an increase in IL6 levels but no difference in TNF α in preeclampsia while Olusi et al; [7] found increased levels in both IL6 and TNF α in normal pregnancy instead. The disparity in the reports from these studies may be from differences in time of sampling, studies not distinguishing between mild or severe forms of preeclampsia and differences in study design; prospective or cross sectional as opposed to case control studies, amongst others. The Ozier [16] study was a cross sectional study involving women at different gestational ages, the study design plus the relatively small sample size used may explain the difference in the findings of this group. In the Afshari study, [8] some of the patients were on dexamethasome therapy and this may have affected the levels of the inflammatory cytokines. The Olusi [7] study did not discriminate between mild and severe disease. In our study, the cases and controls were appropriately matched for maternal age and gestational age.

The finding of significantly increased levels of IL 6 and TNF α and CRP in the women with severe preeclampsia from our study was corroborated by findings from a meta-analysis by Cui Xie et al; [17] who concluded that their findings strengthened the clinical evidence that preeclampsia was accompanied by exaggerated inflammatory responses. A systematic review and meta-analysis by Sien Yee Lau et al; [18] which included also, non parametric data analysis reported increased IL6 and TNF α levels in preeclampsia.

The cytokines, IL6 and TNF α, are proteins produced principally by activated lymphocytes and macrophages but also by endothelial, epithelial and connective tissues that function by modulating the function of other cell types. TNF α and IL 6 mediate immunologic, inflammatory and reparative host responses [19]. In preeclampsia, the ischemic placenta may contribute to the maternal endothelial cell dysfunction by enhancing the synthesis of IL6, TNF α and IL8 [1]. Studies using animal models have shown that increasing cytokine levels during pregnancy, raises blood pressure and decreases renal function. The pathological change in the kidney is glomerulosclerosis. The glomeruli show solid appearance with great narrowing or occlusion of the tuft. The capillary endothelium is swollen due to oedema with thickening of the capillary basement membrane and widening of the interstitial spaces with intense oedema. The underlying mechanisms of hypertension in pregnancy in response to increased cytokine levels appear to involve activation of endothelin 1, increased oxidative stress, and activation of angiotensin II type 1 receptors. [19, 20]. TNF-α have been shown to directly stimulate endothelial cells in culture to secrete endothelin 1 and cell adhesion molecules which would attract leukocytes to adhere to vascular tissues and play a role in edema and hypertension. [21]. TNF α induces oxidative damage as it destabilizes electron flow in the mitochondria resulting in the release of oxidizing free radicals and formation of peroxides that damage endothelial cells [22]. TNF α stimulates angiotensin II production in the female reproductive tract and IL6 up regulates the expression of angiotensin II type 1 receptors in vascular smooth muscles [23].

High levels of IL 6, and TNF α induce the systemic acute phase response stimulating the liver to synthesize CRP [24]. C-reactive protein has been shown to be a sensitive marker of tissue damage and inflammation [25]. This study also reports significant differences in the plasma levels of creatinine and uric acid, and the liver enzymes, aspartate amino transferase (AST), alanine amino transferase (ALT), alkaline phosphatise (ALP) and gamma glutamyl transferase (GGT) between pre-eclamptic subjects and control group.

Preeclampsia is a multisystem disorder with greatest effects on the central nervous system, the liver, the kidney and the coagulation system [1], with resultant organ damage [26]. The increased plasma levels of these markers above the reference limits for the healthy pregnant population is indicative of some degree of organ dysfunction [27, 28].

Severe pre-eclampsia and eclampsia cause hepatocellular dysfunction reflected by elevation of serum transaminases and/or alkaline phosphatise [29]. Grossly, the liver shows diffuse, fine or blotchy haemorrhages over the capsule or on cut surface, while histologically, fibin thrombi are found in portal vessels and hepatocellular necrosis [30].

This study reports a significant relationship between CRP and increasing blood pressure, uric acid, ALP and AST suggesting that inflammation is involved in the tissue damage that occurs in preeclampsia. The association of CRP with disease severity in preeclampsia has been reported by other authors [31–34]. Duricic et al; [31] reported a negative association between CRP and weeks of gestation in women with preeclampsia, Ali et al; [32] reported an association between CRP and mean arterial pressure in preeclampsia. A cross sectional study by Savvidou et al; [33] did not find increased levels of CRP in a prospective study of pregnant women who later developed preeclampsia but the study by Tjoa et al; [34] where the cases and the controls were matched for maternal age, gestational age, parity and gravidity, reported increased levels of CRP in the first trimester blood samples of women who later developed preeclampsia, suggesting a role for CRP as a predictive marker for preeclampsia in early pregnancy.

The reduced uterine perfusion pressure and resultant placental ischemia in preeclampsia induces the release of cytokines that mediate immunologic, inflammatory and reparative host responses that contribute to the widespread endothelial damage and resultant hypertension and organ dysfunction in preeclampsia [1, 19].

## Conclusion

A role has been suggested for inflammatory mediators in the pathogenesis and pathology of preeclampsia. This study reports increased levels of the inflammatory cytokines, IL6, TNF α and the marker of inflammation, CRP in severe preeclampsia. This study also reports an association between CRP and markers of vascular and organ damage in severe preeclampsia. There may be a place for anti inflammatory agents in the management of women who develop preeclampsia.

## References

[1] Reslan OM, Khalil RA (2010). Molecular and vascular targets in the pathogenesis and management of the hypertension associated with preeclampsia. Cardiovasc Hematol Agents Med Chem..

[2] Wegmann TG, Lin H, Guilbert L, Mosmann TR (1993). Bidirectional cytokine interactions in the maternal-fetal relationship: is successful pregnancy a Th2 phenomenon?. Immunol Today..

[3] Soeters PB, Grimble RF (2013). The conditional role of inflammation in pregnancy and cancer. Clin Nut..

[4] Saito S, Shiozaki A, Nakashima A, Sakai M, Sasaki Y (2007). The role of the immune system in preeclampsia. Mol Aspects Med..

[5] Robertson SA, Bromfield JJ, Tremellen KP (2003). Seminal ‘priming’ for protection from pre-eclampsia-a unifying hypothesis. J Reprod Immunol..

[6] Lai Z, Kalkunte S, Sharma S (2011). A Critical Role of Interleukin-10 in Modulating Hypoxia-Induced Preeclampsia-Like Disease in Mice. Hypertension..

[7] Olusi SO, Diejomaoh M, Omu A, Abdulaziz A, Prabha K, George S (2000). Interleukins in preeclampsia. Annals of Saudi Medicine..

[8] Afshar JT, Ghomian N, Shameli A, Shakeri M, Fahmidehkar MA, Mahajer E (2005). Determination of Interleukin-6 and Tumor Necrosis Factor-alpha concentrations in Iranian-Khorasanian patients with preeclampsia. BMC Pregnancy and Childbirth..

[9] Romero-Adrián T, Ruiz A, Molina-Vílchez R, Estévez J, Atencio R (2002). Interleukin-2 receptor serum concentrations in normal pregnancy and pre-eclampsia. Invest Clin..

[10] Greer IA, Lyall F, Perera T, Boswell F, Macara LM (1994). Increased concentrations of cytokines interleukin-6 and interleukin-1 receptor antagonist in plasma of women with preeclampsia: a mechanism for endothelial dysfunction?. Obstet Gynaecol..

[11] (2000). Report of the National High Blood Pressure Education Program Working Group on High Blood Pressure in Pregnancy. Am J Obstet Gynecol..

[12] Brown MA, Lindheimer MD, de Swiet M, Van Assche A, Moutquin JM (2001). The classification and diagnosis of the hypertensive disorders of pregnancy: statement from the International Society for the Study of Hypertension in Pregnancy (ISSHP). Hypertens Pregnancy..

[13] Wild D (1996). Immunoassay Handbook.

[14] Teran E, Escudero C, Moya W, Flores M, Vallance P (2001). Elevated C-reactive protein and pro-inflammatory cytokines in Andean women with pre-eclampsia. Int J of Gynecol and Obst..

[15] Hentschke MR, Lucas LS, Krauspenhar B, Raisa PF, Sussela AO, Berlesi F (2014). Increased levels of the soluble receptor of Interleukin-6 in patients with preeclampsia compared to normotensive pregnant women. Scientia Medica..

[16] Ozler A, Turgut A, Sak ME, Evsen MS, Soydinc HE, Evliyaoglu O (2012). Serum levels of neopterin, tumor necrosis factor-alpha and Interleukin-6 in preeclampsia: relationship with disease severity. Eur Rev Med Pharmacol Sci..

[17] Cui Xie, Mian Zhi Yao, Jiang Bo Liu, Li Kuan Xiong (2011). A meta-analysis of tumor necrosis factor-alpha, interleukin-6, and interleukin-10 in preeclampsia. Cytokine..

[18] Lau SY, Guild S-J, Barrett CJ, Chen Q, McCowan L, Jordan V, Chamley LW (2013). Tumor necrosis factor-alpha, interleukin-6 and interleukin-10 levels are altered in preeclampsia: a systematic review and meta-analysis. Am J Reprod Immunol..

[19] Abbus A, Lichtman A (2005). Cellular and Molecular Immunology. General Properties of the Immune Response, Cells and Tissues of the Immune System.

[20] Conrad KP, Benyo DF (1997). Placental cytokines and the pathogenesis of preeclampsia. Am J Reprod Immunol..

[21] Cid MC, Kleinman HK, Grant DS, Schnaper HW, Fauci AS, Hoffman GS (1994). Estradiol enhances leukocyte binding to tumor necrosis factor (TNF)-stimulated endothelial cells via an increase in TNF-induced adhesion molecules E-selectin, intercellular adhesion molecule type 1, and vascular cell adhesion molecule type 1. J Clin Invest..

[22] Conrad KP, Miles TM, Benyo DF (1998). Circulating levels of immunoreactive cytokines in women with preeclampsia. Am J Reprod Immunol..

[23] Wesselman JP, De Mey JG (2002). Angiotensin and cytoskeletal proteins: role in vascular remodeling. Curr Hypertens Rep..

[24] Hansson GK (2005). Inflammation, atherosclerosis, and coronary artery disease. N Engl J Med..

[25] Tuomisto K, Jousilahti P, Sundvall J, Pajunen P, Salomaa V (2006). C-reactive protein, interleukin-6 and tumor necrosis factor alpha as predictors of incident coronary and cardiovascular events and total mortality: a population-based, prospective study. Thromb Haemost..

[26] Roberts JM (1998). Endothelial dysfuncion in preeclampsia. Semin Reprod Endocrinol..

[27] Stillman IE, Karumanchi SA (2007). The glomerular injury of preeclampsia. J Am Soc Nephrol..

[28] Munazza B, Raza N, Naureen A, Khan SA, Fatima F, Ayub M, Sulaman M (2011). Liver function tests in preeclampsia. J Ayub Med Coll Abbottabad..

[29] Udenze IC, Arikawe AP, Azinge EC, Egbuagha EU (2014). Liver function tests in Nigerian women with severe preeclampsia. J Clin Sci..

[30] Rolfes DB, Ishak KG (1986). Liver disease in toxaemia of pregnancy. Am J Gastroenterol..

[31] Duricic S, Stojanov M, Obradovic I, Glicic A, Plecas D (2003). Fibronectin and c-reactive protein in pregnancy induced hypertension. Jugoslov Med Biohem..

[32] Ali MA, Hameed BH, Kamel WM (2012). The association of serum cancer antigen 125 and c – reactive protein level with the severity of preeclampsia. Karbala J Med..

[33] Savvidou MD, Lees CC, Parra M, Hingorani AD, Nicolaides KH (2002). Levels of C-reactive protein in pregnant women who subsequently develop pre-eclampsia. BJOG..

[34] Tjoa ML, van Vugt JM, Go AT, Blankenstein MA, Oudejans CB, van Wijk IJ (2003). Elevated C-reactive protein levels during first trimester of pregnancy are indicative of preeclampsia and intrauterine growth restriction. J Reprod Immunol..

